# Neutrophils and Neutrophil Extracellular Traps in Cardiovascular Disease: An Overview and Potential Therapeutic Approaches

**DOI:** 10.3390/biomedicines10081850

**Published:** 2022-08-01

**Authors:** Kohsuke Shirakawa, Motoaki Sano

**Affiliations:** Department of Cardiology, Keio University School of Medicine, Shinjuku-ku, Tokyo 1608582, Japan; shirakawa19840905@z6.keio.jp

**Keywords:** neutrophils, cardiovascular diseases, hydrogen gas

## Abstract

Recent advances in pharmacotherapy have markedly improved the prognosis of cardiovascular disease (CVD) but have not completely conquered it. Therapies targeting the NOD-like receptor family pyrin domain containing 3 inflammasome and its downstream cytokines have proven effective in the secondary prevention of cardiovascular events, suggesting that inflammation is a target for treating residual risk in CVD. Neutrophil-induced inflammation has long been recognized as important in the pathogenesis of CVD. Circadian rhythm-related and disease-specific microenvironment changes give rise to neutrophil diversity. Neutrophils are primed by various stimuli, such as chemokines, cytokines, and damage-related molecular patterns, and the activated neutrophils contribute to the inflammatory response in CVD through degranulation, phagocytosis, reactive oxygen species generation, and the release of neutrophil extracellular traps (NETs). In particular, NETs promote immunothrombosis through the interaction with vascular endothelial cells and platelets and are implicated in the development of various types of CVD, such as acute coronary syndrome, deep vein thrombosis, and heart failure. NETs are promising candidates for anti-inflammatory therapy in CVD, and their efficacy has already been demonstrated in various animal models of the disease; however, they have yet to be clinically applied in humans. This narrative review discusses the diversity and complexity of neutrophils in the trajectory of CVD, the therapeutic potential of targeting NETs, and the related clinical issues.

## 1. Introduction

In recent years, inflammation has gained attention as a residual risk in cardiovascular disease (CVD), and it is being investigated in proof-of-concept clinical trials. In clinical practice, the NOD-like receptor family pyrin domain containing 3 (NLRP3) inflammasome-interleukin (IL)-1β-IL-6 high-sensitivity C-reactive protein (hsCRP) pathway has been a therapeutic target against inflammation [1]. Starting with the JUPITER (Justification for the Use of Statin in Prevention: An Intervention Trial Evaluating Rosuvastatin) trial, which showed that—in addition to low-density lipoprotein cholesterol (LDL-C) levels—hsCRP levels can also be a marker for predicting the reduction of cardiovascular event risk with statin therapy [2], the CANTOS (Canakinumab Antiinflammatory Thrombosis Outcome Study) showed that canakinumab, a human anti-IL-1β monoclonal antibody, is effective in the secondary prevention of myocardial infarction (MI) [3]. Subsequently, in COLCOT (Colchicine Cardiovascular Outcomes Trial) and the LoDoCo2 (Low-dose Colchicine 2) trial, colchicine, an inhibitor of the NLRP3 inflammasome, was reported to be effective in the secondary prevention in patients with coronary artery disease (CAD) [4,5]. The ZEUS (Ziltivekimab Cardiovascular Outcomes Study) is currently underway to determine whether ziltivekimab, a human monoclonal antibody that is directed against the IL-6 ligand, is effective in preventing major adverse cardiovascular events (defined as a composite of nonfatal stroke, nonfatal MI, and cardiovascular death) in patients at a high cardiovascular risk who have chronic kidney disease and elevated hsCRP [6].

Neutrophils are immune cells that form the first line of defense against viral, bacterial, fungal, and parasitic infections. They do so through degranulation, phagocytosis, the production of reactive oxygen species (ROS), and the construction of neutrophil extracellular traps (NETs), i.e., web-like structures that are composed of neutrophil DNA with antimicrobial proteins, including neutrophil elastase (NE), myeloperoxidase (MPO), and cathepsin G, histones, and granules [7,8]. Neutrophils are not only essential for defense against infection but also are extensively involved in the pathogenesis of diseases, from inflammation induction to tissue repair [9]. Although neutrophils had been recognized as a homogeneous population, accumulated evidence clearly demonstrates that they acquire diversity in a disease-specific manner and that not only quantitative but also qualitative changes in neutrophils influence cardiovascular outcomes.

In this narrative review, we summarize some recent basic and clinical findings on how the multifaceted trait alterations in neutrophils and NET formation are involved in the pathogenesis of inflammation in CVD. We also highlight the feasibility and challenges of developing anti-inflammatory therapies for CVD that target NETs.

## 2. Life Cycle of Neutrophils

### 2.1. Neutrophil Release from the Bone Marrow

Neutrophils account for approximately 70% of circulating leucocytes and, in humans, more than 1 × 10^11^ to 2 × 10^11^ neutrophils are produced daily in the bone marrow (BM) [10]. Neutrophils are mobilized from the BM physiologically or in response to stress conditions, and this mobilization involves the antagonistic action of CXC chemokine receptor 2 (CXCR2) and CXCR4 on the neutrophil surface [11].

In the hematopoietic stem cell niches of BM, neutrophil adhesion to endothelial and stromal cells is mediated via very late antigen-4 (VLA-4)/vascular cell adhesion molecule 1 (VCAM-1) signaling, and this adhesion is enhanced by the CXCR4/CXC ligand 12 (CXCL12) pathway [11,12,13,14,15]. CXCL12 is mainly expressed in osteoblasts [16,17]. During the differentiation process, neutrophils change into CXCR4^+^CXCR2 pre-proliferating neutrophils, CXCR4^-^CXCR2^low^ non-proliferating immature neutrophils, and finally, CXCR2^high^ neutrophils. The decreased expression of CXCR4 allows the release of neutrophils from the BM into the circulatory system [12]. The CXCR2 ligands, CXCL1 and CXCL2, are expressed on osteoblasts and endothelial cells in BM, and CXCR2/CXCR2 ligand signaling stimulates neutrophil egress from the BM [11,14]. Of note, the hematopoietic stem cell niche is regulated by the sympathetic nervous system: Noradrenaline that is released by sympathetic nerves acts on the β3-adrenergic receptors of stromal cells to inhibit CXCL12 expression, thereby promoting neutrophil outflow into the circulation [18,19].

### 2.2. Aging of Neutrophils in Circulating Blood

Neutrophils newly that are released from the BM disappear from the circulating blood in as little as 6 to 12 h, during which time they undergo intrinsic changes, termed neutrophil senescence, that follow circadian patterns [20,21,22,23]. In mice, fresh neutrophils that are produced in the BM are released into the circulation during the night when the mice are active, whereas the aged neutrophils are removed from the peripheral circulation during the daytime, when the mice are inactive [24]. Diurnal changes in phenotypes include the loss of CD62L (also known as L-selectin) and an increase in CXCR4 expression [24].

In mice, the diurnal rhythm of neutrophils is regulated by the clock gene *Arntl*, which encodes Bmal1; Bmal1 triggers CXCR2-dependent transcriptional changes by regulating the circadian expression of CXCL2 and driving cell-autonomous neutrophil phenotypic and functional changes, namely the aging program [22]. In contrast, CXCR4 antagonizes CXCR2-mediated signaling and thereby blocks the aging program [22].

The “aged” neutrophils are mainly returned to the BM, a process that is thought to be facilitated by the re-expression of CXCR4 on the neutrophils [25,26,27,28]. They are then eliminated by macrophages in the BM [29].

### 2.3. Homeostatic Function of Neutrophils

Recent evidence suggests that after their lifespan in the circulating blood, neutrophils also enter healthy tissues other than the BM and help to maintain tissue homeostasis [22]. Most infiltrate the spleen and lungs, but some infiltrate other organs, such as the intestines, heart, adipose tissue, and kidneys [24,25].

In general, neutrophils eliminate invading pathogens through a variety of inflammatory responses, including degranulation, phagocytosis, ROS production, and the release of NETs [8]. The contents of neutrophil granules are cytotoxic and include MPO, cathepsin G, elastase 2, cathelicidin antimicrobial peptide, and matrix metalloproteinase 8 (MMP8), and neutrophils generate a large amount of ROS by activating superoxide-generating enzyme nicotinamide adenine dinucleotide phosphate (NADPH) oxidase, a process that is known as the ″oxidative burst. Neutrophils phagocytose pathogens, then the neutrophil granules fuse with the pathogen-containing phagosomes, and subsequently, ROS and neutrophil proteases work together to kill the ingested pathogens. The NETs that are produced by neutrophils have highly adhesive properties, so they trap pathogens, which are then eliminated by the various types of NET granules [7].

Curiously, despite their abundance of cytotoxic granules and ability to produce ROS, neutrophils rarely cause tissue damage when they are mobilized into normal tissues. Adrover et al. reported that neutrophils that are freshly released from the BM contain abundant granules and are capable of NET formation, but while flowing in the peripheral blood, they degranulate by a mechanism that is regulated by the clock protein Bmal1 and CXCR2 and their NET-forming capacity is also reduced. This cell-intrinsic disarmament program causes aging neutrophils to have blunted inflammatory properties, protecting tissues from excessive inflammation [21]. However, another report showed the opposite, i.e., enhanced ROS production, adhesion, and phagocytosis by aged human neutrophils [30]. Therefore, further studies are needed to determine how the inflammatory properties of neutrophils are altered during neutrophil aging in physiological and pathological settings.

### 2.4. Leukocyte Adhesion Cascade

Circulating neutrophils are mobilized to inflammatory foci through a process that is called the leukocyte adhesion cascade [31]. The activated vascular endothelial cells adjacent to inflammatory sites express adhesion receptors such as P-selectin and E-selectin, which mediate leukocyte rolling on the activated endothelium. Efficient effector function of neutrophils requires β2 integrins [32]. When integrins bind to ligands such as intercellular adhesion molecule-1 (ICAM-1) and ICAM-2 on endothelial cells, neutrophils can firmly adhere and migrate to peripheral tissues [33]. The expression of CXCL1 and CXCL2 by resident cells is essential for neutrophil recruitment to inflammatory sites [34]. At the inflammatory sites, various stimuli, such as cytokines, chemokines, pathogen-associated molecular patterns, and damage-associated molecular patterns, cause neutrophils to undergo tissue-specific phenotypic changes [20].

### 2.5. NET Formation

The dysregulation of neutrophil processes, including the overproduction or defective degradation of NETs, can cause excessive inflammation and the development of potentially fatal pathologies, including acute respiratory distress syndrome and immunothrombosis [35,36]. As discussed below, NETs are also deeply involved in CVD.

NET formation can be triggered by a variety of microbial stimuli and inflammatory mediators. In general, it requires the production of ROS, large amounts of which are produced and released by neutrophils during the oxidative burst [36,37]. ROS-dependent activation of protein arginine deiminase 4 (PAD4) promotes citrullination of histones, nuclear membrane loss, and the release of NET components [36]. NET formation is defective in NE knockout mice and MPO-deficient patients, showing that NE and MPO—proteins that are derived from azurophil granules—are essential for NET formation [38,39]. Activation of the NRLP3 inflammasome in macrophages induces NET formation via the production of IL-1β and IL-18 [40]. Conversely, NETs activate the NRLP3 inflammasome in macrophages. Histones that are derived from NETs directly interact with toll-like receptor 2 (TLR2) that is expressed on T-cells, leading to phosphorylation of signal transducer and activator of transcription (STAT) 3 and differentiation of T-cells into T helper 17 (Th17) cells, which are capable of mobilizing high numbers of neutrophils [41,42,43]. In this way, NETs amplify neutrophil-centered inflammation.

## 3. Neutrophils in CVD

Neutrophils are primed by various lifestyle-related disease risk factor stimuli, such as hyperglycemia, and the activated neutrophils contribute to sterile inflammatory responses such as atherosclerosis and the wound healing processes after MI through degranulation, phagocytosis, generation of ROS, and the release of neutrophil extracellular traps (NETs) [44]. In particular, NETs promote immunothrombosis through their interaction with vascular endothelial cells and platelets and have been implicated in the pathogenesis of various types of CVD, including acute coronary syndrome (ACS), deep vein thrombosis, and heart failure [45].

### 3.1. Neutrophil Counts as Prognostic Factors in CVD

In a cohort study of 12 types of CVD in a sample of more than 700,000 patients, patients with a neutrophil count in the upper limit of the normal range had higher incidences of unheralded coronary death, nonfatal MI, heart failure, peripheral artery disease, and abdominal aortic aneurysm than those with a neutrophil count at the lower end of the normal range [46]. Another study found that increased neutrophil counts are comparable to hypertension as a risk factor for CVD [47]. Furthermore, neutrophil counts correlate with recurrent ischemic events and mortality in CAD [48,49]. An elevated neutrophil to lymphocyte ratio is associated with CAD mortality, left atrial thrombosis, and impaired myocardial perfusion [47,50,51]. In an analysis of data from the Jackson Heart Study, a study of African American people living in Jackson, Mississippi, USA, a higher neutrophil to lymphocyte ratio was prospectively associated with all-cause mortality, coronary heart disease, and heart failure [52]. However, the causal relationship between the ratio and CVD remains unclear. Further investigations are also required to assess how an increase in the absolute number of neutrophils or qualitative changes in neutrophils affect the pathogenesis of CVD.

### 3.2. Effects of Cardiovascular Risks on Neutrophil Activation

Hyperglycemia purportedly increases the risk of developing CVD by generating primed inflammatory neutrophils. In a diabetic mouse model, hyperglycemia promoted myelopoiesis and neutrophil activation in the BM [53]. In hyperglycemic conditions, the neutrophil-derived S100 calcium-binding proteins A8/A9 (S100A8/A9) stimulated hepatic Kupffer cells via the receptor for advanced glycation end products, resulting in increased IL-6 production. IL-6 acts on the hepatocytes to stimulate thrombopoietin production, causing inflammatory platelet hyperplasia [54]. Increasing the levels of IL-6 are also associated with cardiovascular events [55]. The activated Kupffer cells are involved in amplifying inflammation and may be a novel therapeutic target in CVD. In mice with high-fat diet-induced obesity, elastase that was released from azurophil granules of neutrophils was found to disrupt insulin receptor substrate 1 in adipocytes and hepatocytes, mediating insulin resistance and lipogenesis [56].

Hyperglycemia promotes NET formation both in vitro and in vivo by activating NADPH oxidase, which increases ROS production [57,58]. NETs are associated with diabetic complications such as diabetic retinopathy and delayed healing [57,59,60]. The neutrophils of patients with Type 1 and 2 diabetes show elevated expression of peptidyl arginine deiminase 4 (PAD4), an important enzyme for histone citrullination and chromatin decondensation [60].

Angiotensin II (Ang II), a vasoconstrictor, plays an important role in hypertension and atherosclerosis. In addition, it induces ROS production in neutrophils, resulting in NET formation, and the Ang II receptor Type I antagonist losartan suppresses NET formation [61].

Hyperglycemia and the activation of the renin-angiotensin system may increase the risk of CVD by causing changes in the neutrophil phenotype. Future studies are needed to investigate whether lifestyle modifications such as weight loss and exercise, as well as the administration of glucose-lowering drugs and renin-angiotensin system inhibitors, can ameliorate changes in the inflammatory phenotype of neutrophils.

### 3.3. Neutrophils in the Post-MI Heart

Neutrophils are the first responders that amplify the acute inflammatory response after MI. At the onset of MI, the sympathetic nervous system is activated, and signaling at β3 adrenergic receptors in the BM decreases CXCL12 expression in the hematopoietic stem cell niche, which mobilizes neutrophils into the circulating blood [18]. Damage-associated molecular patterns that are released from necrotic cardiomyocytes activate neutrophils, and the activated neutrophils remove the damaged tissue. Neutrophils secrete vascular endothelial growth factor and also play an important role in angiogenesis in injured tissues [62]. However, the persistent infiltration of neutrophils leads to delayed inflammation resolution and tissue repair, resulting in adverse left ventricular remodeling [63]. After fulfilling their role, neutrophils undergo apoptosis and are subsequently ingested by macrophages [64].

Recently, the activation of gasdermin D (GSDMD) in neutrophils at the early stage of MI was found to play an important role in the increased production of neutrophils and their mobilization to the infarcted lesion [65]. GSDMD is a pore-forming protein and is required for the formation and release of NETs [66,67]. During NET formation, GSDMD is proteolytically activated by neutrophil proteases. Cleaved and activated GSDMD forms a pore in the granule membrane, facilitating the release of proteases into the cytoplasm, which leads to further GSDMD activation. These events allow proteases to migrate into the nucleus, where they process histones and enable nuclear expansion. Furthermore, in the final step, the activated GSDMD forms a pore in the plasma membrane, allowing the release of NETs. In mice, genetic deletion of GSDMD reduces neutrophil/monocyte recruitment to the infarcted heart, resulting in reduced infarct size, improved cardiac function, and increased survival after MI [65]. However, the involvement of NETs in this process is unknown.

Neutrophil polarity at the site of MI changes over time [68]. In the early phase (day 1 after MI), the inflammatory neutrophils (known as N1) infiltrate the heart, and on days 5 and 7, the anti-inflammatory neutrophils (known as N2) promote healing by polarizing macrophages to a repair phenotype [9,69]. Recent single-cell analyses have revealed that neutrophils follow different aging trajectories in the circulating blood and in ischemic myocardium [70]. Neutrophils infiltrating the heart immediately after the onset of MI, on day 1, had the same gene expression profile as BM-derived juvenile neutrophils. However, from day 3 onward, a subset of SiglecF^hi^ neutrophils emerged that were not present in the circulating blood and whose gene expression profile suggested that they had increased effector function (phagocytosis, ROS production). This subset of SiglecF^hi^ neutrophils was considered to be a locally acquired phenotype within the microenvironment of the MI lesion.

**Figure 1:**
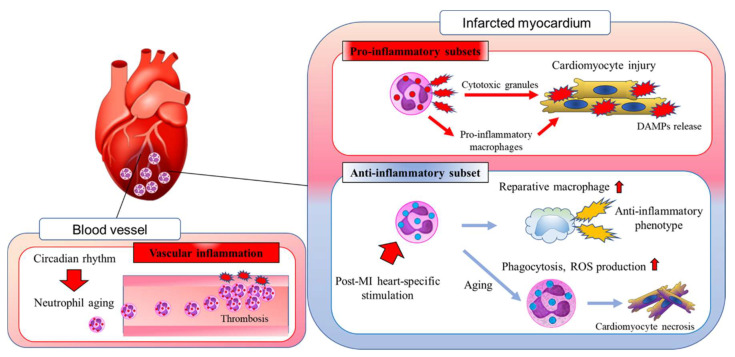
In response to myocardial infarction (MI), neutrophils migrate into the damaged myocardium. In the post-MI heart, neutrophils amplify cardiac inflammation by releasing cytotoxic granules and stimulating the differentiation of proinflammatory macrophages. During the wound healing phase, the heart-specific microenvironment induces neutrophil aging, which results in the resolution of cardiac inflammation. Neutrophils also contribute to the differentiation of alternatively activated reparative macrophages in the post-MI heart. Neutrophil aging is regulated by the circadian rhythm, but the local microenvironment also has a marked effect on cardiovascular homeostasis. DAMPs, damage-associated molecular patterns; MI, myocardial infarction; ROS, reactive oxygen species.

Thus, fresh neutrophils migrating from the BM undergo an aging process that is specific to the post-MI heart and distinct from the physiological aging process that is regulated by the circadian rhythm (Figure 1). The elimination of inflammatory neutrophils after MI or the promotion of differentiation of anti-inflammatory tissue-repairing neutrophils may be considered as a therapeutic strategy to preserve cardiac function after MI. The detailed mechanisms of the disease- and organ-specific phenotypic changes in neutrophils remain unclear, but their elucidation may help prevent adverse cardiac remodeling post-MI.

### 3.4. Neutrophil Aging Process and MI Outcome

In a mouse model of acute MI, due to left anterior descending artery ischemia-reperfusion, the extent of myocardial damage showed diurnal variation, with a larger infarct size at zeitgeber time 5 (5 h after the lights were turned on, i.e., in the phase when mice are inactive), when the circulating aged neutrophils are present. Consistent with this, the infarct size was larger in the Cxcr4ΔN mutant mice, which had more aged neutrophils in the peripheral blood, and smaller in the ArntlΔN mutant mice, in which the circulating neutrophils remain fresh and were not aged [22]. The authors concluded that the aged neutrophils increase the infarct size by promoting the formation of immunothrombi in the blood vessels. Neutrophils that are circulating in the peripheral blood degranulate through a proteome release process that is mediated by CXCR2-CXCL2 signaling [21]. As CXCR4 acts antagonistically with CXCR2, one would expect degranulation to be enhanced in Cxcr4ΔN mutants. Thus, at first glance it appears to be contradictory that during ischemia-reperfusion injury, the disarmed aging neutrophils, which should have low NET-forming capacity, promote intravascular thrombus formation. However, it is quite possible that the local functional and morphological changes of neutrophils during aseptic tissue injury are different from the processes that follow circadian rhythms in normal conditions. Indeed, in ex vivo systems, we found that DNA damage by phorbol-12-myristate-13 acetate (PMA) promotes NET induction, resulting in increased MPO granules within neutrophils via the increased expression of CXCR4 on the cell surface. In support of this finding, we observed that more PMA-stimulated dsDNA is released in the granule-rich early morning neutrophils than in the granule-poor daytime neutrophils [71]. CXCR4 expression on neutrophils in the inflammatory setting may inhibit the physiological disarming process by CXCR2-CXCL2 signaling and contribute to increased NET formation.

### 3.5. Mechanistic Insight into Immunothrombosis Induced by NETs

**Figure 2:**
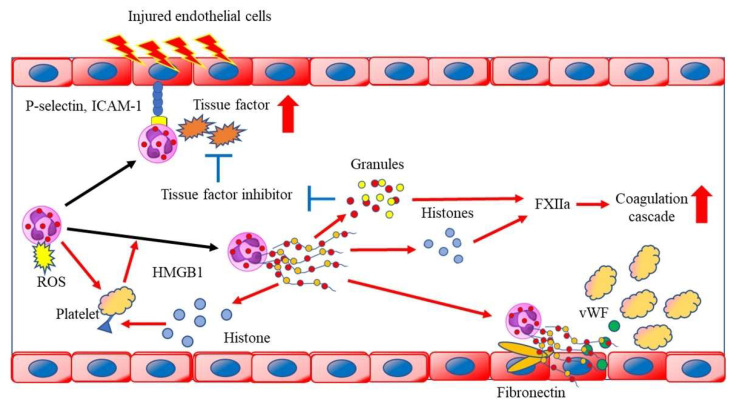
Neutrophils activate platelets through the production of reactive oxygen species (ROS) and the activated platelets present high mobility group box 1 to neutrophils and induce neutrophil extracellular trap (NET) formation. NETs bind to FXIIa and tissue factor (TF), causing the activation of the coagulation cascade. Neutrophil-derived granules degrade the TF pathway inhibitors. Extracellular histone proteins lead to thrombin production and platelet activation via toll-like receptor 2 (TLR2) and TLR4, enhancing immunothrombosis. DNA that is released from NETs also serves as a scaffold for binding neutrophil granule proteins, von Willebrand factor, and fibronectin, a process that promotes platelet binding. HMGB1, high mobility group box 1; ICAM-1, intercellular adhesion molecule 1; ROS, reactive oxygen species; vWF, von Willebrand factor.

Neutrophils and neutrophil-derived inflammatory mediators have been implicated in immunothrombosis (Figure 2). Studies of laser-induced endothelial injury in mice showed that neutrophils infiltrate injured endothelial cells before platelets do, promoting platelet activation, the coagulation cascade, and fibrin generation [72]. The neutrophils activate platelets through the production of ROS [73], and the activated platelets present high mobility group box 1 (HMGB1) to the neutrophils, inducing NET formation [74]. The NETs bind to active coagulation factors, such as factor XII and tissue factor, causing the activation of the coagulation cascade and the generation of thrombin and fibrin [75,76]. Neutrophil granules, such as cathepsin G and elastase 2, degrade tissue factor pathway inhibitor, thereby promoting coagulation [75]. The extracellular histone proteins lead to thrombin production and platelet activation via TLR2 and TLR4, enhancing immunothrombosis [77]. Furthermore, NETs bind fibrinogen and promote fibrin deposition and fibrin network formation [78]. DNA that is released from NETs also serves as a scaffold for binding neutrophil granule proteins, von Willebrand factor (vWF), and fibronectin, a process that promotes platelet binding [79]. Neutrophils that are isolated from MI foci are highly activated and form aggregates with platelets [80]. Both granular proteins and ROS that are released by neutrophils activate endothelial cells, and these activated cells further induce neutrophils in the injured tissue [81]. Hence, neutrophils are increasingly recognized as being important in thrombosis formation and as a risk factor for CVD; however, therapies that target neutrophils to suppress immunothrombosis have not been implemented.

### 3.6. NETs in Coronary Artery Disease

After MI, large numbers of neutrophils accumulate in coronary arteries and contribute to coronary thrombosis by forming NETs, the primary scaffold for platelets, red blood cells, and fibrin [80]. In patients with ST-segment elevation MI (STEMI), the levels of NETs-related factors, such as dsDNA and MPO/DNA complexes, are significantly higher in the coronary blood than in the peripheral blood. Elevated coronary dsDNA levels may predict in-hospital major adverse cardiovascular events more sensitively than creatine kinase muscle and brain and troponin T [82]. Furthermore, in patients with STEMI, the extent of NET formation in coronary thrombosis correlated positively with the area under the curve of creatine kinase muscle and brain and infarct size that was measured by cardiac magnetic resonance and negatively with ST segment resolution on electrocardiogram [80]. In a cohort of 30 patients with STEMI and stable angina who underwent successful percutaneous coronary intervention, a gradual decrease in dsDNA was reported in all the patients 14 days after the intervention, although during the observation period the dsDNA levels remained higher in patients with STEMI than in those with stable angina [83]. NETs have also been implicated in the pathophysiology of stent thrombosis [84]. MPO levels correlate with the prevalence and severity of CVD [85,86,87]. A clinical study examining whether double-stranded DNA and MPO DNA in the circulating blood are associated with clinical outcomes and increased coagulability in patients with stable CAD found that only the double-stranded DNA levels were significantly associated with adverse clinical outcomes at 2 years (unstable angina, nonbleeding stroke, MI, and death) [88]. Men, smokers, patients with metabolic syndrome, and patients with previous MI had significantly higher double-stranded DNA levels; however, the association between the double-stranded DNA levels and hypercoagulability was weak. Double-stranded DNA is not necessarily derived solely from NETs. Further studies with more specific NETs markers (e.g., histone citrullination of neutrophils) are warranted.

### 3.7. NETs in Atherosclerotic Plaques

In animal models, several neutrophil-derived inflammatory factors, such as IL-37, heparin-binding protein (HBP/CAP37/azurocidin), MMP8, and ROS, are implicated in the development and instability of atherosclerotic plaques. IL-37 and heparin-binding protein (HBP/CAP37/azurocidin) specifically stimulate the mobilization of inflammatory monocytes via the activation of formyl peptide receptors [89]. During the development of atherosclerosis, infiltrated monocytes differentiate into macrophages, which contribute to plaque formation as lipid-rich foam cells [90]. MMP8 degrades collagen and induces plaque instability [91]. ROS activates endothelial cells, leading to neutrophil mobilization and activation, and oxidizes LDL, which may contribute to plaque vulnerability [92].

NETs have been identified in human atherosclerotic foci, and the presence of NETs indicates a more severe atherothrombotic state [93,94]. In human atherosclerotic plaques, neutrophils and NETs are seen near clusters of apoptotic endothelial and smooth muscle cells that contribute to plaque disruption [95,96]. The rupture of the fibrous capsule of atherosclerotic plaques has been considered as the primary cause of ACS, and there is evidence for the involvement of NETs in plaque rupture [74]. However, advances in imaging techniques, such as optical coherence tomography, now indicate that erosion of the plaque while it retains its fibrous capsule is responsible for about one-third of cases of ACS [97]. NETs seem to be involved in the process of plaque erosion. The process begins with a change in the endothelial shear stress gradient, which activates TLR2 on the endothelial cells, leading to a loss of basement membrane integrity and shedding of endothelial cells, followed by the formation of NETs and thrombosis [95]. Cholesterol crystals activate IL-1β transcription in response to macrophages and simultaneously activate inflammasomes and stimulate IL-1β secretion. NETs act synergistically with cholesterol crystals in the transcriptional activation of IL-1β in macrophages. The secreted IL-1β upregulates the T-cell-derived cytokine IL-17 and drives CXCL1 and CXCL2 to amplify neutrophil recruitment to atherosclerotic plaques [98]. NETs are also triggered by P-selectin, which is expressed by the activated platelets or the activated endothelium [99]. NETs components, such as cathelicidin antimicrobial peptide and cathepsin G, can induce the migration of monocytes and macrophages into a plaque [100,101]. After plaque rupture, the interaction between neutrophils and thrombin-activated platelets at the injured lesion further promotes NET formation [76].

As mentioned above, evidence is accumulating that NETs are involved in both plaque rupture and erosion, but the effect of NETs on atherosclerotic plaque formation itself is still unclear [96,102]. In an atherosclerosis model in which low-density lipoprotein receptor-deficient mice were fed a high-fat diet, the loss of Pad4 in hematopoietic cells by BM transplantation to suppress NET formation had no effect on fatty streak and plaque formation [96].

In summary, although the contribution of NETs to the formation of atherosclerotic plaques is debatable, they appear to play an important role in the development of ACSs that are associated with plaque rupture and erosion. In addition to anticoagulation and antiplatelet therapies, clinical trials assessing whether neutrophil-targeted anti-immunothrombotic therapies are effective in preventing or treating ACS may be worthwhile.

### 3.8. NETs in Venous Thrombosis

Venous thrombosis is usually initiated by endothelial dysfunction [103]. Endothelial cells contain Weibel-Palade bodies (WPBs), granules that store vWF, P-selectin, and other vasoregulatory factors; in response to vascular injury, the endothelial cells secrete WPBs, which release vWF. Secreted as ultra-high molecular weight multimers, vWF adheres to both platelets and collagen in subendothelial tissue that is exposed at the site of vascular injury. P-selectin is translocated from within the WPBs to the extracellular surface and is released by exocytosis, and externalized P-selectin that is anchored to the plasma membrane interacts with a leukocyte ligand, P-selectin glycoprotein ligand-1. Some platelets that are bound to vWF multimers are activated, express P-selectin, and adhere to neutrophils [99], and the platelet-neutrophil interaction promotes the formation of NETs [104]. Histones in NETs stimulate the release of WPBs from vascular endothelial cells [105], forming a feedforward loop.

In a mouse model of deep venous thrombosis that was induced by flow restriction of the inferior vena cava, venous thrombi contained large amounts of NET components, such as the extracellular citrullinated histone H3, in the vicinity of vWF [106]. Genetic depletion of PAD4 and intravenous administration of deoxyribonuclease 1 ameliorated thrombus formation [106,107]. However, in humans, thromboses that were recovered from patients with venous thrombosis contained significantly fewer NETs than coronary thromboses from patients with MI [80]. Further studies are needed on the involvement of NETs in venous thrombosis and their potential as therapeutic targets in humans.

### 3.9. NETs in Heart Failure

Although the pathogenesis of heart failure is heterogeneous and involves many different types of inflammation, recent evidence suggests that NETs are involved in chronic cardiac dysfunction. In mice, Ang II-induced cardiac hypertrophy and dysfunction were alleviated by neutrophil depletion with anti-Ly6G antibodies, whereas they were exacerbated by acute neutrophilia that was achieved by neutrophils transfusion [108]. Mechanistically, Ang II induced neutrophil activation, resulting in adhesion to the vessel wall and NET formation. NETs triggered microthrombosis and the impairment of myocardial microcirculation. Chronic microthrombosis leads to capillary rarefaction, further exacerbating myocardial hypoxia and impairing cardiac function. Leukocytes from human and murine models of heart failure were characterized by markedly decreased expression of Kruppel-like factor 2 (*Klf2*), a suppressor of myeloid inflammatory activation. The downregulation of *Klf2* enhances the inflammatory phenotype of neutrophils via the activation of hypoxia-inducible factor 1 and other factors, activating the NET-thrombosis pathway [108]. Currently, the main approach to treating heart failure is to reduce the hemodynamic burden on the heart by inhibiting the excessive activation of neurohumoral factors. NET-mediated immunothrombotic mechanisms are promising new therapeutic targets for heart failure.

## 4. Neutrophils in CVD Complicated by COVID-19

CVD is the most common comorbidity in patients with COVID-19 and has been associated with adverse outcomes [109]. Cardiovascular complications due to SARS-CoV-2 infection include ACS, venous thrombosis and pulmonary embolism (associated with abnormal coagulation), and myocarditis [110]. Neutrophil-induced inflammation and NET-induced immunological thromboembolism may contribute to the pathophysiology of COVID-19–related CVD. Indeed, NET formation by circulating and infiltrating neutrophils is increased in patients with COVID-19 [111] and correlates with the clinical severity and prognosis in these patients [112]. The oxidative burst that is associated with SARS-CoV-2 infection stimulates NET formation [113], and the spike proteins and viral RNA of SARS-CoV-2, together with the proinflammatory cytokines TNFα and IL-8, can activate neutrophils. When SARS-CoV-2 or its components adhere to platelets, they promote platelet activation and aggregation and induce NET formation [114,115]. The cytotoxicity of histones that are released from NETs promotes endothelial dysfunction and death [116]. The experience of SARS-CoV-2 infections during the COVID-19 pandemic is reaffirming the universal importance of neutrophils and NETs in the pathophysiology of CVD.

### 4.1. ACS

Excess NET formation that is induced by SARS-CoV-2 infection is important for the etiology of ACS that are associated with COVID-19 [117]. In fact, in a case series of five patients with STEMI that was associated with COVID-19, co-expression of MPO-DNA complexes and citrullinated histone H3, both markers of NETs, was found in the thrombi aspirated from coronary arteries in all the patients [117]. The coronary thrombogenic mechanism involving NETs is a distinct feature of ACS in general. Although coronary thrombosis may be induced by multiple mechanisms, such as platelet activation by SARS-CoV-2 and endothelial dysfunction, NETs formation is undoubtedly the main cause of coronary thrombosis and may be a therapeutic target.

### 4.2. Venous Thrombosis and Pulmonary Embolism

The incidence of venous thromboembolism in patients with COVID-19 is estimated to be around 7.4% to 16.5% [118,119]; this incidence appears to be higher than in other viral infections, suggesting that a thrombogenic mechanism that is specific to COVID-19 is involved. SARS-CoV-2 infects cells via angiotensin-converting enzyme 2 (ACE2), which is expressed on the vascular endothelial cells [120]. ACE2 plays an important role in vascular endothelial cell homeostasis by converting Ang II to Ang 1–7 and antagonizing Ang II-induced ROS production and consequent vascular endothelial injury [121]. SARS-CoV-2 impairs ACE2 function, leading to an increase in oxidative stress in the vascular endothelial cells and progressive endothelial cell injury [122]. This process causes vascular endothelial cells to release vWF, which binds to platelets; vWF stabilizes blood coagulation factor VIII and activates the coagulation cascade, resulting in thrombosis formation [123]. In addition, NET induction via ACE2 receptors on neutrophils and the subsequent activation of the coagulation cascade may also be involved in the development of venous thrombi.

### 4.3. Myocarditis

Acute myocarditis has been reported as a complication of COVID-19. In a study of 1597 U.S. athletes that were infected with SARS-CoV-2 (mean age, 19 years), cardiac magnetic resonance imaging scans revealed that 37 patients (2.3%) had asymptomatic or mild myocarditis [124]. In addition, among 148 patients with COVID-19 with elevated cardiac troponin, 26% of patients had a myocarditis-like pattern on cardiovascular magnetic resonance examination imaging [125]. In 22 patients with COVID-19 who showed elevated troponin, cardiac pathological findings showed no cases of the interstitial lymphocytic infiltrates or extensive myocardial necrosis that are characteristic of common viral myocarditis [126]. NETs have been detected in the endocardium of patients with myocarditis [127]. In addition, in an experimental myocarditis model of mice with coxsackie virus B3 infection, NET production peaked one week after infection, and neutrophil-specific PAD4 knockout reduced cardiac necrosis and inflammation. These findings suggest that NET production that is induced by viral infection is important for the pathogenesis of myocarditis [128]. Although no studies have clarified the extent to which NETs are involved in myocarditis in patients with COVID-19, various cytokines and chemokines that are released during SARS-CoV-2 infection may induce NET formation and lead to the onset of myocarditis.

The cytokine midkine, which is important for neutrophil adhesion during acute inflammation and subsequent extravasation, promotes neutrophil recruitment and NET formation in the heart and contributes significantly to the pathology of myocarditis [127]. In patients with COVID-19, the serum midkine levels are elevated about three-fold compared with healthy controls [129]. It has been suggested that midkine may increase the affinity of SARS-CoV-2 for ACE2 receptors and promote viral invasion and accumulation [130]. These results suggest that midkine and other inflammatory cascades in the pathogenesis of COVID-19 may contribute to the development of myocarditis by promoting NET formation.

### 4.4. Antithrombotic Therapy in Patients with COVID-19

Anticoagulation with low molecular weight heparin for the prevention of thrombosis has been shown to improve survival in patients with COVID-19 without increasing the risk of serious bleeding, so prophylactic anticoagulation with low molecular weight heparin is recommended in all hospitalized patients with COVID-19 who do not have any contraindications [131,132]. Recently, thrombosis prophylaxis with rivaroxaban was reported to have a beneficial effect on clinical outcomes in patients with COVID-19 who have a high thrombotic risk [133]. However, despite prophylactic anticoagulation, the incidence of venous thrombosis remains high in COVID-19 [134], suggesting that thrombogenic mechanisms are involved that cannot be prevented by existing strategies. Although the risk of exacerbation of infection due to suppression of NET production must be considered, the suppression of neutrophil activation and NET production may help to improve vascular endothelial damage and immunothrombosis and may reduce the prevalence of CVD that is associated with COVID-19.

## 5. Potential Therapeutics Targeting NETs

Given that NET formation that is associated with neutrophil activation is deeply involved in the pathogenesis of CVD, NET formation and NETs themselves may be promising therapeutic targets for the prevention and treatment of various types of CVD [82]. Some potential therapeutics are discussed below.

### 5.1. PAD4 Blockade and DNase I

Both PAD4 blockade and Dnase I-driven removal have been evaluated as promising NET-targeting therapeutic strategies. In apolipoprotein E-deficient mice that *were* fed a high-fat diet, the PAD inhibitor Cl-amidine blocked NET formation, decreased the size of the atherosclerotic lesion, and delayed carotid thrombosis onset time upon photochemical injury [135]. In acute carotid injury experiments, hematopoietic PAD4 deficiency or DNase I treatment protected mice from plaque erosion [96]. In a rat model of myocardial ischemia-reperfusion injury, a combination of DNase I and recombinant tissue-type plasminogen activator reduced NET formation and improved coronary microvasculature flow, leading to a reduction in infarct size and left ventricular remodeling [136].

However, in sterile inflammation the suppression of NETs has not only advantages but also disadvantages. DNase therapy may release highly active enzymes and toxic molecules such as histones that can damage healthy cells [137]. Furthermore, NETs exhibit anti-inflammatory effects such as degradation of cytokines and chemokines [138]. NETs also promote macrophage polarization toward a reparative phenotype. In fact, the wound healing process after MI is impaired in NET-deficient mice [139]. In addition, as stated above, NETs are important as a defense mechanism against infection. In mouse models of acute lung injury that was induced by bacteria such as methicillin-resistant *Staphylococcus aureus* and *Pseudomonas aeruginosa*, the partial deletion of PAD4 (heterozygous PAD4-deficient mice) and DNase I treatment had a beneficial effect on attenuating the lung injury without negatively affecting the bacterial burden and also improved survival; in contrast, the complete loss of PAD4 (homozygous PAD4-deficient mice) slightly reduced the lung injury and reduced bacterial clearance but ultimately did not improve survival [140]. Even in sterile inflammation, the possibility of an increased risk of infection should always be considered when targeting NETs.

### 5.2. Th2 Cytokines

So-called Type 2 immune responses, in which Th2 cytokines such as IL-4 and IL-13 play a central role, are involved in the pathogenesis of atopic and allergic diseases. Interestingly, patients with allergic diseases such as atopic dermatitis have fewer skin neutrophils than people with healthy skin and are more susceptible to bacterial skin infections [141], and the MPO levels and NET formation capacity are known to be reduced in neutrophils from patients with allergic diseases compared with those from healthy individuals [142]. Experimental evidence indicates that when the Type 2 cytokines IL-4 and IL-13 predominate, signaling via the Type 2 IL-4 receptors on neutrophils suppresses neutrophil effector functions such as mobilization, chemotaxis, and NET formation. Type 2 IL-4 receptor-mediated signaling suppresses neutrophil recruitment from the BM by decreasing CXCR2 expression and increasing CXCR4 expression via the activation of signal transducer and activator of transcription 6 and p38 mitogen-activated protein kinases. It also inhibits neutrophil chemotaxis by activating p38 mitogen-activated protein kinases and thus directly inhibiting the CXCR2-phosphoinositide 3-kinase pathway. Further studies are needed to determine how Th2 cytokines affect the aging process that is associated with the circadian rhythm of neutrophils in the peripheral blood and the qualitative changes of neutrophils in the inflammatory setting.

### 5.3. Molecular Hydrogen

Recently, we demonstrated the therapeutic potential of molecular hydrogen (H_2_) to inhibit neutrophil activation and NET formation [71]. In various pathological conditions, H_2_ has been experimentally and clinically proven to have therapeutic effects by scavenging hydroxyl radicals and improving inflammation [143,144,145,146,147,148,149,150]. In our previous study, we reported that inhalation of H_2_ during a percutaneous coronary intervention in patients with STEMI reduces left ventricular remodeling at 6 months [150]. We also confirmed the positive effects of H_2_ inhalation on brain damage after cardiopulmonary resuscitation in rats [151]. A multicenter randomized clinical trial on the clinical application of H_2_ inhalation in post-cardiac arrest syndrome is ongoing in Japan [152].

A published multicenter randomized clinical trial demonstrated that H_2_ inhalation improves symptoms and prognosis in patients with moderate COVID-19 pneumonia [153]. As NETs have been implicated in both the severity of COVID-19 pneumonia and in left ventricular remodeling after MI, we sought to test the hypothesis that H_2_ could inhibit excessive neutrophil activation and NET formation. In vitro experiments on human neutrophils revealed that H_2_ can suppress both PMA-induced (ROS-dependent) and Ca^2+^ ionophore-induced (ROS-independent) neutrophil aggregation and subsequent NET formation [71]. We found that histone H2AX, a DNA damage marker, is present in hyperactivated neutrophils and induces CXCR4 expression as part of the DNA damage response. The upregulation of CXCR4 inhibits homeostatic degranulation by CXCR2, leading to the accumulation of neutrophil granules and creating a situation that facilitates NET formation. Consistent with this finding, NET-forming neutrophils were reported to highly express CXCR4 on their surface [154]. The effects of H_2_ on DNA damage in stimulated neutrophils included the suppression of CXCR4 expression, maintenance of constant degranulation, and the lowering of the NET formation threshold. In vivo, H_2_ inhalation also suppressed NETs formed in the pulmonary arteries of lipopolysaccharide-induced sepsis models of aged micro-miniature pigs [154]. Thus, H_2_ may exert anti-NET effects by various mechanisms, such as the elimination of ROS, the suppression of Ca^2+^-dependent PAD4 activation, the inhibition of neutrophil aggregation, and the suppression of pathogenic cellular senescence of neutrophils.

**Figure 3:**
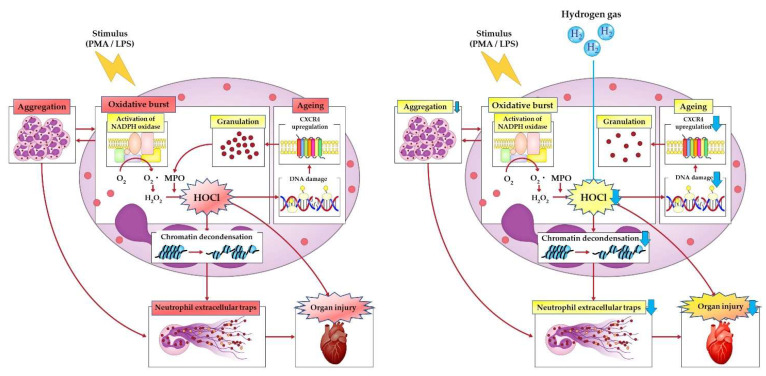
Hydrogen gas causes reduced neutrophil aggregation. Hydrogen gas-mediated neutralization of hypochlorous acid that is produced by the oxidative burst suppresses DNA damage, which is followed by a decrease in CXC chemokine receptor 4 expression on neutrophils. Maintaining the proper disarming process with hydrogen gas leads to reduced citrullination of histones and decreased release of neutrophil extracellular trap (NET) components, making hydrogen gas therapy a potential therapeutic strategy for cardiovascular disease that involves neutrophil inflammation and NET formation. CXCR4, CXC chemokine receptor 4; H_2_O_2_, hydrogen peroxide; HOCl, hypochlorous acid; LPS, lipopolysaccharide; MPO, myeloperoxidase; NADPH, nicotinamide adenine dinucleotide phosphate; PMA, phorbol-12-myristate-13acetate.

Neutrophil activation also has beneficial effects in sterile inflammation. In experimental acute sterile lung injury in mice, alveolar neutrophils acquire the ability to take up and degrade extracellular DNA fragments and allow optimal organ repair only when they are exposed to an inflammatory environment [155]. Furthermore, neutrophils are said to be able to transport matrix from normal to damaged organs to promote wound healing [156]. Consequently, the development of treatments targeting neutrophils in aseptic inflammation must also consider their anti-inflammatory and tissue repair functions. The same is true for treatments targeting NETs. Furthermore, treatments that use DNase I to lysate NETs that have already formed and been released may instead risk causing systemic inflammation by spreading NET contents, such as histones and MPO, throughout the body. H_2_ inhibits neutrophil aggregation and NET formation by PMA-stimulated human neutrophils without affecting gene expression profiles or phagocytosis and chemotaxis. Consequently, H_2_ therapy is expected to be effective in the prevention and treatment of NET-related CVD because it can potently inhibit the formation only of excessive NETs on activated neutrophils without affecting the essential tissue homeostatic function of neutrophils (Figure 3).

## 6. Conclusions

Although neutrophils have a very short lifespan, their functional dynamism and localization are tightly regulated by circadian rhythms, which explains the clinical observation that disease severity varies with the time of day. In addition, in inflammatory milieu neutrophils undergo disease- and organ-specific phenotypic changes, which have a profound impact on the outcome of reversible and sometimes irreversible organ damage. Understanding the life cycle of neutrophils and the dynamic changes in their function in the local inflammatory setting may lead to the discovery of new targets for the prevention and treatment of CVD.

Neutrophil-mediated inflammation and thrombosis are among the common mechanisms underlying CVD, and the moderate suppression of neutrophil overactivation and NET formation is expected to improve the pathogenesis of various types of CVD, including ACS, thromboembolism, atherosclerosis, heart failure, and myocarditis. In particular, inhalation of H_2_ can efficiently prevent excessive NET formation without suppressing the basic functions of neutrophils. Consequently, H_2_ can reduce chronic inflammation and irreversible organ damage and is expected to have a therapeutic effect not only in infectious diseases but also in various types of CVD. One important strategy to overcome inflammation as a residual risk in CVD will be to develop neutrophil-targeted, NET-suppressive therapies that can be implemented clinically.

## Data Availability

Data sharing not applicable.
